# Exosome-derived lnc-HOXB8-1:2 induces tumor-associated macrophage infiltration to promote neuroendocrine differentiated colorectal cancer progression by sponging hsa-miR-6825-5p

**DOI:** 10.1186/s12885-022-09926-1

**Published:** 2022-08-27

**Authors:** Xiaojun Li, Qiusheng Lan, Wei Lai, Heng Wu, Heyang Xu, Kai Fang, Zhonghua Chu, Yujie Zeng

**Affiliations:** 1 Department of Gastrointestinal Surgery, Sun Yat-Sen Memorial Hospital, Sun Yat-Sen University, Guangzhou, China; 2 Guangdong Provincial Key Laboratory of Malignant Tumor Epigenetics and Gene Regulation, Sun Yat-Sen Memorial Hospital, Sun Yat-Sen University, Guangzhou, China

**Keywords:** Colorectal cancer, Neuroendocrine differentiation, Exosome, lnc-HOXB8-1:2, Tumor-associated macrophage

## Abstract

**Introduction:**

Neuroendocrine differentiation (NED) in colorectal cancer (CRC) cells has been known for decades, and our previous meta-analysis indicated that CRC patients with neuroendocrine differentiation have a lower 5-year survival rate. In recent years, an increasing number of studies have found that exosome-derived long non-coding RNAs (lncRNAs) play important roles in cancer progression and metastasis. However, the functions and mechanism of exosome-derived lncRNAs in CRC with neuroendocrine differentiation are not yet fully clear.

**Materials and methods:**

The clinical significance of NED was assessed in a retrospective study of 105 patients. Next-generation sequencing and bioinformatics analysis were conducted to select lnc-HOXB8-1:2 for further study. Using immunohistochemistry, qRT–PCR, western blot, transwell assay, immunofluorescence assay, fluorescence in situ hybridization assay and dual-luciferase reporter assay, the oncogenic role of exosome-derived lnc-HOXB8-1:2 was determined in CRC with NED. The mechanism underlying the lnc-HOXB8-1:2/hsa-miR-6825-5p/CXCR3 axis was also explored.

**Results:**

NED was a risk factor for the progression and mortality of CRC. lnc-HOXB8-1:2, derived from exosomes secreted by neuroendocrine differentiated colon cancer cells, was identified in our study. The proportion of M2 macrophages and the migration and invasion capacities of tumor-associated macrophages (TAMs) markedly increased after the addition of neuroendocrine differentiated CRC cell-derived exosomes. More excitingly, the expression of lnc-HOXB8-1:2 and the protein level of CXCR3 were also upregulated in TAMs. The lnc-HOXB8-1:2/hsa-miR-6825-5p/CXCR3 axis was predicted via miRanda software and confirmed by the dual-luciferase reporter assay. Furthermore, the increased expression of lnc-HOXB8-1:2 was accompanied by downregulation of hsa-miR-6825-5p expression and upregulation of CXCR3 protein levels. Overexpression of hsa-miR-6825-5p also reduced CXCR3 expression.

**Conclusion:**

lnc-HOXB8-1:2 in exosomes derived from neuroendocrine differentiated CRC cells acted as a ceRNA competitively binding hsa-miR-6825-5p to upregulate CXCR3 expression and leading to TAM infiltration and M2 polarization, which promotes neuroendocrine differentiated CRC progression.

**Supplementary Information:**

The online version contains supplementary material available at 10.1186/s12885-022-09926-1.

## Introduction

Ranking as the third leading cause of malignancy morbidity, colorectal cancer (CRC) is the second leading cause of death from malignancy worldwide [1, 2]. The pathogenesis and progression mechanism of CRC are not yet fully clear, even though great efforts have been devoted to their elucidation. Neuroendocrine differentiation (NED) in colorectal cancer cells has been known for decades [3]. Neuroendocrine differentiation refers to neuroendocrine cells scattered as either single cell or cell nests within adenocarcinoma in a proportion between 2% and 30%, which can be detected by chromogranin A (CgA) and synaptophysin (Syn) [4, 5]. Our previous study suggested that colon cancer cells with neuroendocrine differentiation induce the infiltration of tumor-associated macrophages (TAMs) by increasing the expression of CXCL10 and CXCL11, thus the proliferation and invasiveness of CRC were intensified [5], while the specific molecular pathway involved have not been elucidated. Additionally, some studies have proven that chemokine CXC motif receptor 3 (CXCR3), the chemokine receptor for CXCL10 and CXCL11, is associated with poor prognosis in CRC [6, 7]. Tumors with high CXCR3 expression are prone to cancer cell proliferation and infiltration, both in CRC and mantle cell lymphoma [8, 9], although the mechanism leading to elevated levels of CXCR3 remains unknown.

Exosomes also serve essential roles in cancer progression and metastasis, including CRC [10]. Released by various types of cells, exosomes are nanosized lipid bilayer membrane extracellular vesicles (30–150 nm) that carry lipids, DNAs, RNAs, proteins and other biomolecules [11]. These cargos are protected from degradation due to the lipid bilayer membrane structure of exosomes [12]. By transferring and exchanging these contents, exosomes participate actively in intercellular communication [13]. Exosomes were found to be secreted at high levels in CRC tissue than in normal colonic mucosa, and the secretion of exosomes differ according to lymph node staging [14]. In addition, recent studies have revealed that many exosome-derived proteins, circRNAs, miRNAs and lncRNAs contribute to tumorigenesis, proliferation, metastasis and treatment resistance in CRC [15–17].

Long non-coding RNAs (lncRNAs), members of the non-coding RNA family, are transcripts of no less than 200 nucleotides with limited ability to translate into proteins [18]. Increasing evidence has shown that lncRNAs exert regulatory functions in various biological processes, including gene expression, growth, differentiation and development, as well as the occurrence and progression of cancers [18–20]. LncRNA-RMRP is highly expressed in bladder cancer and promotes cancer cell proliferation, migration and invasion by competitively inhibiting miRNA-206 [21]. Exosome-derived MALAT1, a lncRNA lacking open reading frames, facilitates the malignant behavior of CRC by acting as a miR-26a/26b sponge to regulate FUT4 and stimulate the PI3K/Akt pathway [17]. Although many lncRNAs have been found to participate in the initiation and progression of cancer, the functions and mechanism of exosome-derived lncRNAs in CRC with neuroendocrine differentiation are still largely unknown.

In the present study, we were the first to demonstrate the identification of lnc-HOXB8-1:2 in exosomes derived from CRC cells with NED and further explored the possible mechanism by which lnc-HOXB8-1:2 alters the malignant traits of CRC based on our preliminary study [5].

## Materials and methods

### Patients

From April 2015 to July 2016, at the Department of Gastrointestinal Surgery of Sun Yat-sen Memorial Hospital, Sun Yat-sen University, we selected 105 patients with CRC who underwent surgery for retrospective analysis. The inclusion criteria were as follows: (1) pathological diagnosis of colorectal adenocarcinoma; (2) the markers of NED, including CgA and Syn, were detected by immunohistochemistry; (3) available histologic samples (i.e., surgically resected specimens); and (4) valid clinical data, including surgery, treatment and follow-up data, which were collected through case reviews and telephone follow-up. The following follow-up data were recorded: current physical condition, date of local recurrence and/or metastasis, date and cause of death, etc. Overall survival (OS) was defined as the interval from surgery to the date of mortality or the final follow-up. Progression-free survival (PFS) was defined as the interval from surgery to the date of recurrence, metastasis, or mortality. The end point was set as July 31, 2021. Patients who died perioperatively (within 3 months after surgery) or received preoperative chemotherapy and/or radiotherapy were excluded.

### Cell culture

The LoVo colon cancer cell line, 293 T cells and THP-1 cells were purchased from the Chinese Academy of Sciences, Shanghai Institutes for Biological Sciences. The LoVo colon cancer cell line was selected to transform into stable CgA neuroendocrine-like cells (LoVo-CgA) and control cells (LoVo-NC) according to the method described previously [5], the CgA vector and NC vector were labeled with green fluorescent protein (GFP) gene. The PCR fragment of lnc-HOXB8-1:2 was inserted into the pcDNA3.1( +) vector, which was used to infect LoVo-CgA cells, in addition to a separate control vector experiment. Lipofectamine 2000 was used for transfection according to the manufacturer’s instruction. Following infection, selective media containing G418 were used until the stable clone cell line (LoVo-CgA-OE) and control cell line (LoVo-CgA-NC) were obtained after 2 weeks. THP-1 cells were treated with 320 nM PMA for 6 h and then 20 ng/mL IL-4 was added for another 18 h to obtain tumor-associated macrophages [5]. TAMs were transfected with hsa-miR-6825-5p mimics as well as the corresponding control oligonucleotides using reagent Lipofectamine RNAimax based on the manufacturer’s instructions. Stable clone LoVo colon cancer cells and 293 T cells were incubated in DMEM-12 supplement with 10% FBS, and the other cells were incubated in RPMI-1640 supplement with 10% FBS at 37 °C with 5% CO_2_. All specific overexpression plasmids were designed and synthesized by General Biosystems Co., Ltd (Anhui, China) and hsa-miR-6825-5p mimics were purchased from GenePharma (Suzhou, China). The details of main reagents were listed in Table S1.

### Coculture of tumor-associated macrophages with exosomes or colon cancer cells

Approximately 1 × 10^6^ tumor-associated macrophages were inoculated in the upper chamber of 6-well plates, and cocultured with LoVo-CgA-OE or LoVo-CgA-NC cells at a ratio of 1:1 or with different exosomes (20 µg/mL) [22] in the lower transwell chamber for 48 h, separately. The tumor-associated macrophage cells were washed for subsequent experiments after coculture.

### Immunohistochemistry (IHC) assay

An IHC assay was used to detect CgA, Syn, CD68 and CXCR3 proteins. The staining procedure was conducted using the Envision two-step method as previously reported [5]. PBS replaced the primary antibody as a negative control. Primary antibodies against CgA (1:500), Syn (1:500), CD68 (1:500) and CXCR3 (1:600) were used. Horseradish peroxidase (HRP)-linked polyclonal rabbit anti-mouse IgG was used as the secondary antibody (1:1000). The details of main antibodies were listed in Table S2.

### Extraction and identification of exosomes

The original culture media of cells was aspirated, and then the cells were washed twice with PBS, and replenished with serum-free culture media for 36 h. The culture media was then harvested. The cell media was centrifuged at 2000 × g for 30 min to remove cells and debris, and the supernatant containing the cell-free culture media was transferred into a new tube without disturbing the pellet. Total Exosome Isolation Reagent (from cell culture media) was used to extract exosomes from different cell culture media according to the manufacturer's instructions. After exosomes were isolated, the morphology of exosomes was observed by transmission electron microscopy (TEM), and the particle size was detected by Zeta View (Particle Metrix). Total RNA and protein of exosomes were purified for subsequent RNA sequencing and western blot assays, respectively, using the Total Exosome RNA and Protein Isolation Kit.

### RNA isolation and quantitative reverse transcription PCR (qRT–PCR) assay

Total RNA from cell lysates was isolated using TRIzol and reverse transcription was conducted using M-MLV Reverse Transcriptase. GoTaq® RT–PCR Master Mix was applied to perform qRT–PCR according to the manufacturer’s protocol to examine the level of mRNAs, miRNAs and lncRNAs. The specific sequences of the qRT–PCR primers were listed in Supplementary Table S3.

### Western blot assay

Cells were collected and lysed in RIPA buffer on ice. The Bradford assay was performed to detect the protein concentration. Equivalent amounts of protein were separated on 10% and 12% SDS–PAGE gels and transferred onto PVDF membranes. Five percent nonfat milk was used to seal the membranes. After blocking, the membranes were incubated at 4 °C overnight with primary antibodies against CgA (1:1000), CD68 (1:1000), CXCR3 (1:1000), CD63 (1:1000), HSPA8 (1:1000), Alix (1:1000) and TSG101(1:1000) and then incubated with HRP-linked polyclonal rabbit anti-mouse IgG (1:2000) for 1 h at room temperature after washing. An enhanced chemiluminescence (ECL) kit was used to visualize the labeled proteins. GAPDH served as an internal reference. The details of main antibodies were listed in Table S2.

### Cell migration and invasion assay

For cell migration assays, single-cell suspensions were diluted to 1 × 10^6^ cells in serum-free RPMI-1640 medium and 100 µL was seeded into the upper transwell chamber, followed by the addition of 600 µL RPMI-1640 medium containing 10% FBS in the lower chamber. The cells were allowed to incubated at 37 °C with 5% CO_2_ in a humidified atmosphere. After 24 h, nonmigrating cells were removed and the other cells were fixed with 4% paraformaldehyde for 10 min, stained with 0.1% crystal violet for 10 min, washed with PBS, and counted in under a microscope. Cell migration assays were also conducted using the same transwell inserts with 10% Matrigel spread onto the upper chamber. The invasive cells were incubated for 48 h. Three random fields were selected for cell counting.

### Next-generation sequencing (NGS)

Total RNA was extracted from exosomes secreted by LoVo-CgA cells (CgA Exo) and LoVo-NC cells (NC Exo) for next-generation sequencing (NGS). NGS and gene differential expression analysis were conducted by Forevergen (Guangzhou, China) to select lncRNAs for subsequent study. The inclusion criteria of lncRNAs were as follows: (1) upregulated expression in CgA Exo; (2) number of miRNA potential binding sites in common with CXCR3 mRNA greater than or equal to three; (3) only one transcript, with length between 200 and 2500 base pairs; (4) multiple top 20 of differentially expressed lncRNAs between CgA Exo and NC Exo; and (5) available to search in the LNCipedia database (https://lncipedia.org/).

### Bioinformatics analysis

The association between the gene expression level of CgA or CXCR3 and the infiltration of macrophages, especially M2 macrophages, in different cancers was determined based on the Timer 2.0 database (http://timer.cistrome.org/). According to competitive endogenous RNA (ceRNA) theory [19], miRanda software (Memorial Sloan-Kettering Cancer Center, USA) was used to predict the miRNAs with the most binding sites that could simultaneously bind to lnc-HOXB8-1:2 and CXCR3 mRNA. RNA22 database (https://cm.jefferson.edu/rna22) and Target Scan database (https://www.targetscan.org) were used to validate this prediction.

### Fluorescence in situ hybridization (FISH) assay

Specific fluorescently labeled lnc-HOXB8-1:2 FISH probes were designed and synthesized by GenePharma Co. (Shanghai, China). The specific sequence of the FISH probe was listed in Supplementary Table S4. Paraffin sections were placed at room temperature for 60 min, soaked in xylene for dewaxing and immersed in graded alcohol for rehydration. Subsequently, the slides were heated in 0.01 M sodium citrate buffer solution (pH 6.0) at approximately 95 °C for 15 min, and treated with proteinase K (20 µg/mL) at 37 °C for 30 min. The slides were then treated with lnc-HOXB8-1:2 probe hybridization solution (2 µM) at 37 °C overnight. Subsequently, the slide was washed with 50% formamide at 42 °C, thoroughly rinsed using 2 × saline sodium citrate buffer (SSC), and blocked in 5% normal goat serum at room temperature for 30 min. The slides were used for subsequent immunofluorescence (IF) assays to detect the expression level and site of CD68 protein in CRC tissue specimens.

### Immunofluorescence (IF) assay

A PKH67 Green Fluorescent Cell Linker Mini Kit was used to detect whether labeled exosomes were absorbed by macrophages [23] according to the manufacturer's protocol.

To determine the proportion of M2 macrophages, treated TAMs were grown to 40%-60% confluence in glass bottom dishes, washed with PBS three times and fixed in 4% paraformaldehyde. The cells were incubated with primary antibodies against CD68 (1:600) and CD206 (1:200) at 4 °C overnight. After washing with PBS, the cells were incubated with Alexa Fluor 488-donkey anti-rabbit IgG secondary antibody (1:500) and Alexa Fluor 546-goat anti-mouse IgG secondary antibody (1:500) at room temperature away from light for 1 h and stained with DAPI for 10 min.

To detect the expression level and site of CD68 proteins in CRC tissue specimens, a primary antibody against CD68 (1:400), and Alexa Fluor 594-goat anti-rabbit IgG secondary antibody (1:500) were used in the IF assay procedure mentioned above. The details of main antibodies were listed in Supplementary Table S2.

The fluorescent cells of the IF assay and FISH assay were observed using a Nikon A1Si laser scanning confocal microscope (Nikon Instruments Inc., Japan). The proportion of fluorescent cells was quantified for data analyses.

### Dual-luciferase reporter assay

Dual-luciferase reporter assay was applied to confirm the direct binding between lnc-HOXB8-1:2 and hsa-miR-6825-5p and to explore whether CXCR3 was the direct target of hsa-miR-6825-5p. The sequence of lnc-HOXB8-1:2 and the 3’UTR sequence of CXCR3 containing the wild-type or mutant binding sites with hsa-miR-6825-5p (Supplementary Table S5) were cloned into the psiCHECK-2 vector including Firefly luciferase gene (Fluc) and Renilla luciferase gene (Rluc), respectively. lnc-HOXB8-1:2 vectors and CXCR3 vectors were cotransfected with NC or has-miR-6825-5p mimics into 293 T cells. The relative values of Fluc and Rluc were measured by Centro LB960 XS3 (Berthold, German) using Dual-Luciferase Reporter Assay Systems.

### Statistical analysis

Statistical analysis and graph generation were performed with GraphPad Prism 8.0 (GraphPad Software, USA) and SPSS 20.0 (IBM Corp, USA). Qualitative data were assessed by Pearson’s chi-square test or the Mann–Whitney U rank-sum test. Student’s t test or Welch’s t test was used to perform statistical analysis for quantitative data between two groups. The Kaplan–Meier method and log-rank test were performed to analyze overall survival and progression-free survival. The associations between risk factors and OS or PFS were quantified by hazard ratios (HRs) and 95% confidence intervals (CIs) using a Cox proportional hazards model. A 2-tailed *P* value < 0.05 was considered statistically significant.

## Results

### The clinical prognostic value of NED in colorectal adenocarcinoma

A total of 105 patients diagnosed with colorectal adenocarcinoma were followed up (Table 1). Among these patients, 18 patients (17.1%) were classified as the NED group, whose clinical information is shown in Table 2, and the remaining 87 (82.9%) were in the non-NED group. Their mean age at inclusion was 60.7 ± 13.0 years (range, 22–89 years). The mean follow-up time was 52.2 ± 22.4 months (range, 4–75 months). At the end point, 32 patients died of CRC, 3 died of other causes, and 5 were lost to follow-up.

**Table 1:** Baseline data of neuroendocrine differentiated colorectal cancer patients

Factors	No. Patients				${\chi }^{2}$/$U$	${\rm P}$
	Total	Percentage (%)	NED	Non-NED		
All patients	105	100.0	18	87		
Gender					1.559^a^	0.212
Male	62	59.0	13	49		
Female	43	41.0	5	38		
Age					724.5^b^	0.498
≤ 50	24	22.9	3	21		
> 50	81	77.1	15	66		
Primary tumor site					0.303^a^	0.582
Colon	58	55.2	11	47		
Rectum	47	44.8	7	40		
Tumor differentiation					736.5^b^	0.593
High	18	17.1	3	15		
Moderate	81	77.1	13	68		
Low	6	5.7	2	4		
TNM stage					724.5^b^	0.603
I	13	12.4	3	10		
II	34	32.4	4	30		
III	42	40.0	7	35		
IV	16	15.2	4	12		
Lymphatic metastasis					0.043^a^	0.836
Positive	56	53.3	10	46		
Negative	49	46.7	8	41		
Simultaneous distant metastasis					0.298^c^	0.585
Yes	16	15.2	4	12		
No	89	84.8	14	75		

*NED* Neuroendocrine differentiation group, *non-NED* Non-neuroendocrine differentiation group

^a^ The chi-square test, ${\chi }^{2}$

^b^ The Mann–Whitney U rank-sum test, $U$

^c^ The chi-square test with Yates’ correction, ${\chi }^{2}$

**Table 2:** The clinical information of 18 colorectal adenocarcinoma patients with neuroendocrine differentiation

No.	Gender	Age	Tumor site	TNM stage	Tumor differentiation	Site of metastasis	Time of metastasis	Follow-up result
1	Male	49	Rectum	IVA	Moderate	Lung	Before the operation	Dead, 27 months survival
2	Female	74	Sigmoid colon	IVA	Poor	Liver	Before the operation	Dead, 6 months survival
3	Male	64	Sigmoid colon	IIB	High	Liver / lung	14/43 months after operation	Dead, 54 months survival
4	Male	56	Sigmoid colon	IIIB	Moderate	None	None	Live, follow-up 72 months
5	Male	77	Rectum	IIIB	Moderate	Local recurrence	9 months after operation	Dead, 12 months survival
6	Male	69	Sigmoid colon	IIIC	Moderate	Local recurrence	8 months after operation	Dead, 26 months survival
7	Male	55	Rectum	IVA	Moderate	Liver / liver, surgical incision	Before the operation/ 6 months after operation	Dead, 17 months survival
8	Male	53	Sigmoid colon	IVA	Moderate	Liver	Before the operation	Dead, 9 months survival
9	Female	74	Ascending colon	I	High	None	None	Live, follow-up 68 months
10	Male	76	Rectum	I	Moderate	None	None	Live, follow-up 67 months
11	Female	77	Transverse colon	IIIB	High	Local recurrence	36 months after operation	Dead, 36 months survival
12	Male	77	Ascending colon	I	Moderate	None	None	Live, follow-up 66 months
13	Female	50	Descending colon	IIIC	Moderate	Liver / local recurrence, lung	2/12 months after operation	Dead, 20 months survival
14	Male	62	Ascending colon	IIIA	Moderate	None	None	Live, follow-up 64 months
15	Male	60	Caecum	IIB	Moderate	None	None	Live, follow-up 61 months
16	Male	77	Rectum	IIB	Moderate	None	None	Live, follow-up 72 months
17	Male	32	Rectum	IIIC	Poor	Liver/ celiac lymph node	2/7 months after operation	Dead,8 months survival
18	Female	68	Rectum	IIB	Moderate	Local recurrence	55 months after operation	Dead,60 months survival

**Fig. 1:**
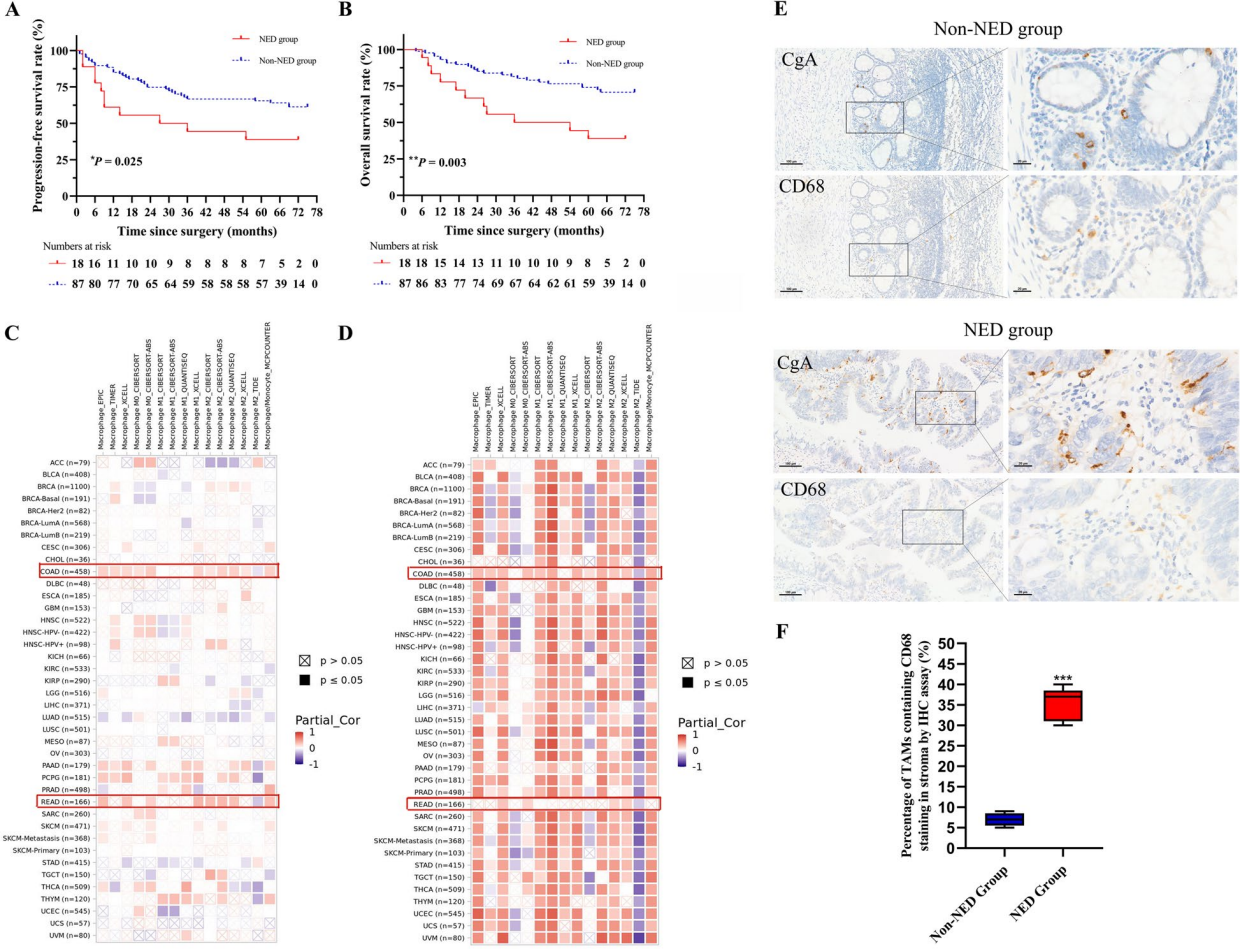
Neuroendocrine differentiation predicted poor prognosis of CRC patients and corelated with macrophage infiltration. **A**, Kaplan–Meier progression-free survival curve of patients in the NED group and the non-NED group. **B**, Kaplan–Meier overall survival curve of patients in the NED group and the non-NED group. **C**, Data from Timer 2.0 database showed the association between CgA gene expression level and macrophage infiltration. **D**, The correlation between CXCR3 gene expression level and macrophage infiltration. **E**, Tumor-associated macrophages (stained by CD68) clustered around neuroendocrine differentiated cells (stained by CgA). Scale bar = 100μm and 20 μm. **F**, The percentage of tumor-associated macrophages containing CD68 staining in the NED group was significantly increased compared to the non-NED group. *, *P* < 0.05; **, *P* < 0.01; ***, *P* < 0.001; NED, neuroendocrine differentiation; non-NED, non-neuroendocrine differentiation; COAD, colon adenocarcinoma; READ, rectal adenocarcinoma; CgA, chromogranin A

Comparing the distributions of clinicopathological factors between the NED group and the non-NED group, there were no significant differences observed (Table 1), indicating that other factors were balanced and comparable between the two groups. Moreover, we found that 11 patients (61.1%) in the NED group experienced local recurrence and/or distant metastasis in the course of CRC, while there were 31 patients (35.6%) in the non-NED group, there was significant difference between the two groups (chi-square test ${\chi }^{2}$=4.304, *P* = 0.045, Table 3). Considering the development of the disease over time, we also compared the progression-free survival (PFS) of the two groups. The 5-year PFS rates of all patients, the NED group and the non-NED group were 61.0%, 38.9% and 65.5%, respectively. In the NED group, 11 patients developed progression during follow-up, the PFS of whom was significantly shorter, with an HR of 2.151 (95% *CI* 1.082–4.275, *P* = 0.029) than that of the non-NED group, in which 32 patients experienced progression (*P* = 0.025, Fig. 1A). In addition, patients in the NED group appeared to have shortened overall survival (*P* = 0.003, Fig. 1B). Eleven patients died before the endpoint in the NED group, and 24 died in the non-NED group. Patients with NED had 2.834 times higher risk (95% *CI* 1.386–5.794, *P* = 0.004) of mortality than those without NED. The 5-year OS rates of all patients, the NED group and the non-NED group were 67.9%, 38.9% and 74.0%, respectively. Namely, colorectal adenocarcinoma with neuroendocrine differentiation appears to have a less favorable prognosis.

**Table 3:** Comparisons of local recurrence and/or metastasis rates between the NED group and the non-NED group

		NED		Total	${\chi }^{2}$	*P*
		Positive	Negative			
Local recurrence or metastasis	Yes	11(61.1%^a^)	31 (35.6%^a^)	42(40.0%^a^)	4.034	0.045^*^
	No	7(38.9%^a^)	56 (64.4%^a^)	63(60.0%^a^)		
Total		18	87	105		

*NED* Neuroendocrine differentiation

^a^ The percentage of column

^*^ Statistical significance (*P* < 0.05)

Furthermore, CXCR3 plays a significant role in immune infiltration in cancer [24]. Using different algorithms, we indicated that the infiltration of macrophages and M2 macrophages was positively associated with the gene expression of CgA (NED marker) in colon adenocarcinoma (COAD) and most rectal adenocarcinoma (READ). Both the infiltration of macrophages and M2 macrophages were proportional to the CXCR3 gene expression level in most colorectal adenocarcinomas, while some results were contrary (Fig. 1C-D). The immunohistochemical sections of colorectal adenocarcinoma from 10 patients (5 from the NED group and 5 from the non-NED group) showed that tumor-associated macrophages (stained by CD68) accumulated where CgA protein was expressed (Fig. 1E). Compared with the non-NED group, the proportion of TAMs clustered around neuroendocrine differentiated cells was significantly increased in the NED group (*P* < 0.001, Fig. 1F).

These results suggested that the poor prognosis of colorectal adenocarcinoma with NED may be due to the high invasiveness, which is possibly associated with the infiltration of macrophages, especially M2 macrophages.

### The identification of neuroendocrine differentiated colon cancer cell-derived exosomes

**Fig. 2:**
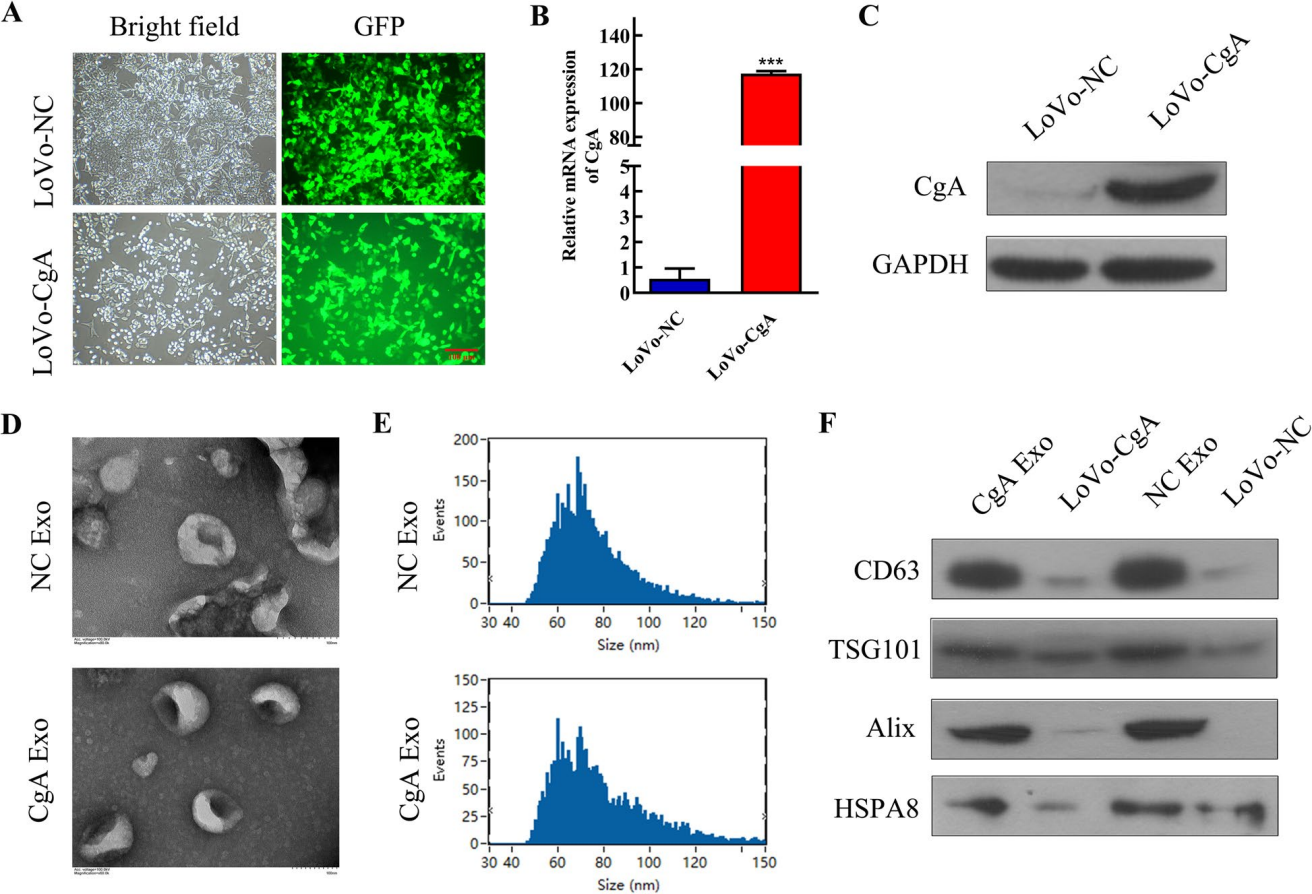
Neuroendocrine differentiated colon cancer cells secrete exosomes. **A**, Fluorograms of stable neuroendocrine differentiated colon cancer line (LoVo-CgA) and negative control cell line (LoVo-NC), green fluorescent proteins (GFP) > 95%. Scale bar = 100 μm. B-C, The mRNA expression and protein expression of CgA was determined in LoVo-NC cells and LoVo-CgA cells using qRT–PCR assay (**B**) and western blot assay (**C**). **D**, The exosomes extracted from LoVo-NC cells (NC Exo) and LoVo-CgA cells (CgA Exo) showed typical cup shapes by transmission electron microscopy (TEM). Scale bar = 100 nm. **E**, The particle size of NC Exo and CgA Exo both ranges from 45 to150nm. **F**, NC Exo and CgA Exo both expressed exosomal labeled protein CD63, TSG101, Alix and HSPA8. ***, *P* < 0.001; NC, negative control; CgA, chromogranin A; Exo, exosomes

Increasing evidence indicats that exosomes play important roles in cancer progression and metastasis [10]. According to our previous study [5], the LoVo colon cancer cell line was selected, and CgA was successfully overexpressed to construct neuroendocrine differentiated colon cancer cells, namely, LoVo-CgA cells (Fig. 2A). A significant elevation in CgA expression was noticed in LoVo-CgA cells by qRT–PCR and western blot analysis (Fig. 2B-C). Exosomes of LoVo-CgA cells and LoVo-NC cells were extracted separately. Both groups of exosomes showed typical cup shapes with particle sizes ranging from approximately 45–150 nm (Fig. 2D-E), which was in line with the general morphological characteristics of exosomes. The exosome-labeled proteins (CD63, TSG101, Alix and HSPA8) were expressed in exosomes isolated from both groups (Fig. 2F).

These findings illustrated that colon cancer cells in vitro have the ability to secrete exosomes, and whether NED affects the prognosis of CRC via exosomes is the object of our enduring curiosity.

### The effect of exosomes from neuroendocrine differentiated colon cancer cells on TAMs

**Fig. 3:**
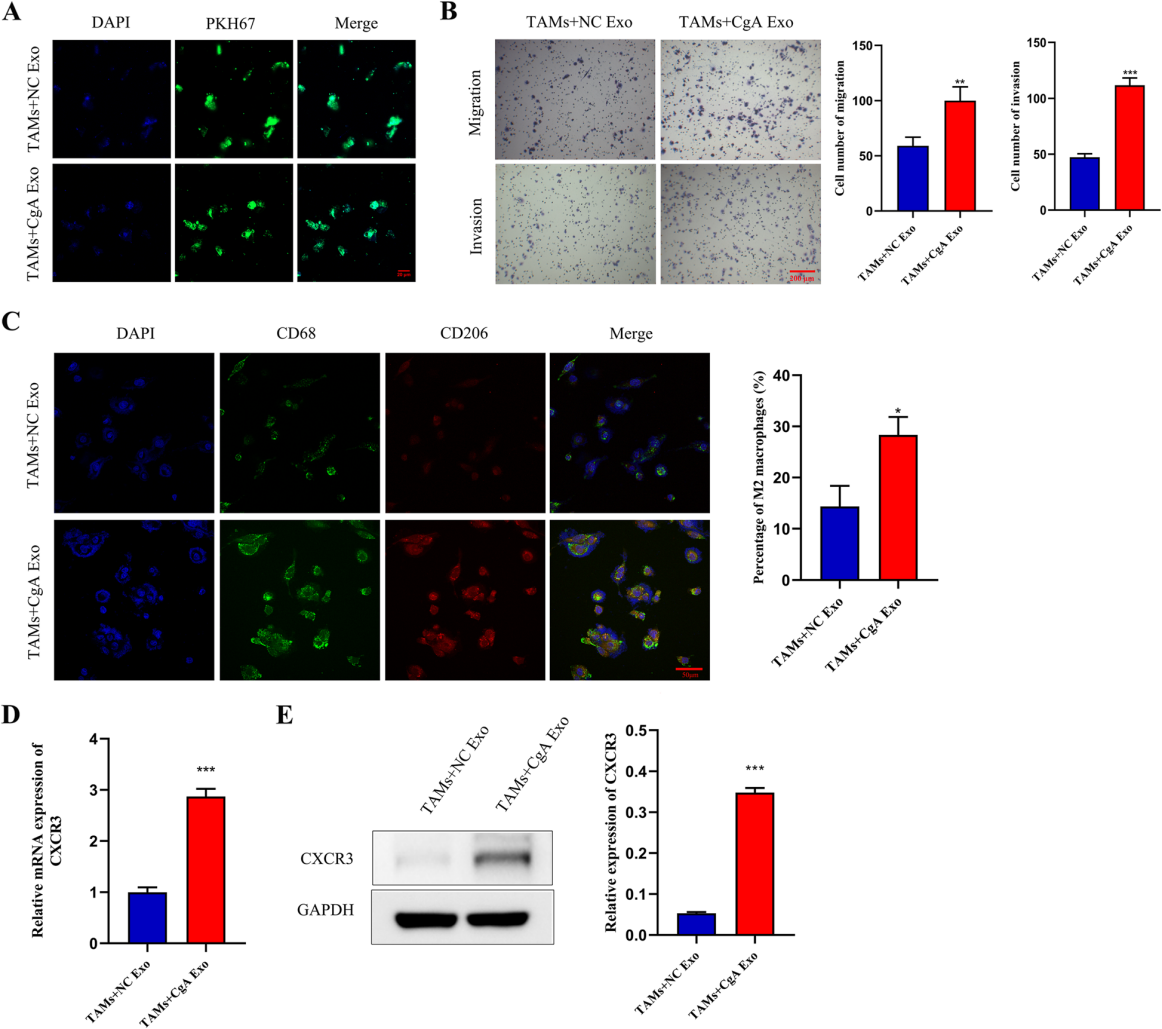
Neuroendocrine differentiated colon cancer cell-derived exosomes enhanced the chemotaxis of TAMs, promoted M2 polarization and upregulated CXCR3 expression. **A**, Exosomes labeled by PKH67 were absorbed by TAMs when they were cocultured with different exosomes. Scale bar = 20 μm. **B**, The cocultured of TAMs and CgA Exo resulted in an obvious improvement of migration and invasion ability of TAMs. Scale bar = 200 μm. **C**, The proportion of M2 macrophages (stained by CD68 and CD206) was determined in TAMs with different treatment by immunofluorescence assay. Scale bar = 50 μm. **D**-**E**, qRT–PCR assay (**D**) and western blot assay (**E**) exhibited that CXCR3 expression in TAMs treated with CgA Exo was elevated. *, *P* < 0.05; **, *P* < 0.01; ***, *P* < 0.001; TAMs, tumor-associated macrophages; NC, negative control; CgA, chromogranin A; Exo, exosomes

Tumor-associated macrophages, one of the most active immune cells, play a pivotal role in the tumor microenvironment. M2 macrophages promote the progression and metastasis of tumors [25, 26]. To explore the effect of exosomes on TAMs, exosomes extracted from LoVo-CgA cells (CgA Exo) and LoVo-NC cells (NC Exo), were cocultured with TAMs differentiated from human THP-1 cells [5], respectively. We found that exosomes labeled with PKH67 were absorbed by TAMs when cocultured (Fig. 3A). Transwell assays revealed that the migration and invasion capacities of TAMs were both increased when cocultured with CgA Exo compared to those treated with NC Exo (Fig. 3B). Furthermore, the proportion of M2 macrophages (stained by CD206) in TAMs cocultured with CgA Exo was significantly higher than that in TAMs cocultured with NC Exo (Fig. 3C). We found that the expression of CXCR3 in TAMs cocultured with CgA Exo was markedly upregulated relative to that in the other group (Fig. 3D-E).

These data showed that exosomes from neuroendocrine differentiated colon cancer cells not only enhance the chemotaxis of TAMs but also promote the differentiation of M2 macrophages, possibly by regulating CXCR3 expression.

### Exosome-derived lnc-HOXB8-1:2 affected TAMs in neuroendocrine differentiated colorectal cancer

**Fig. 4:**
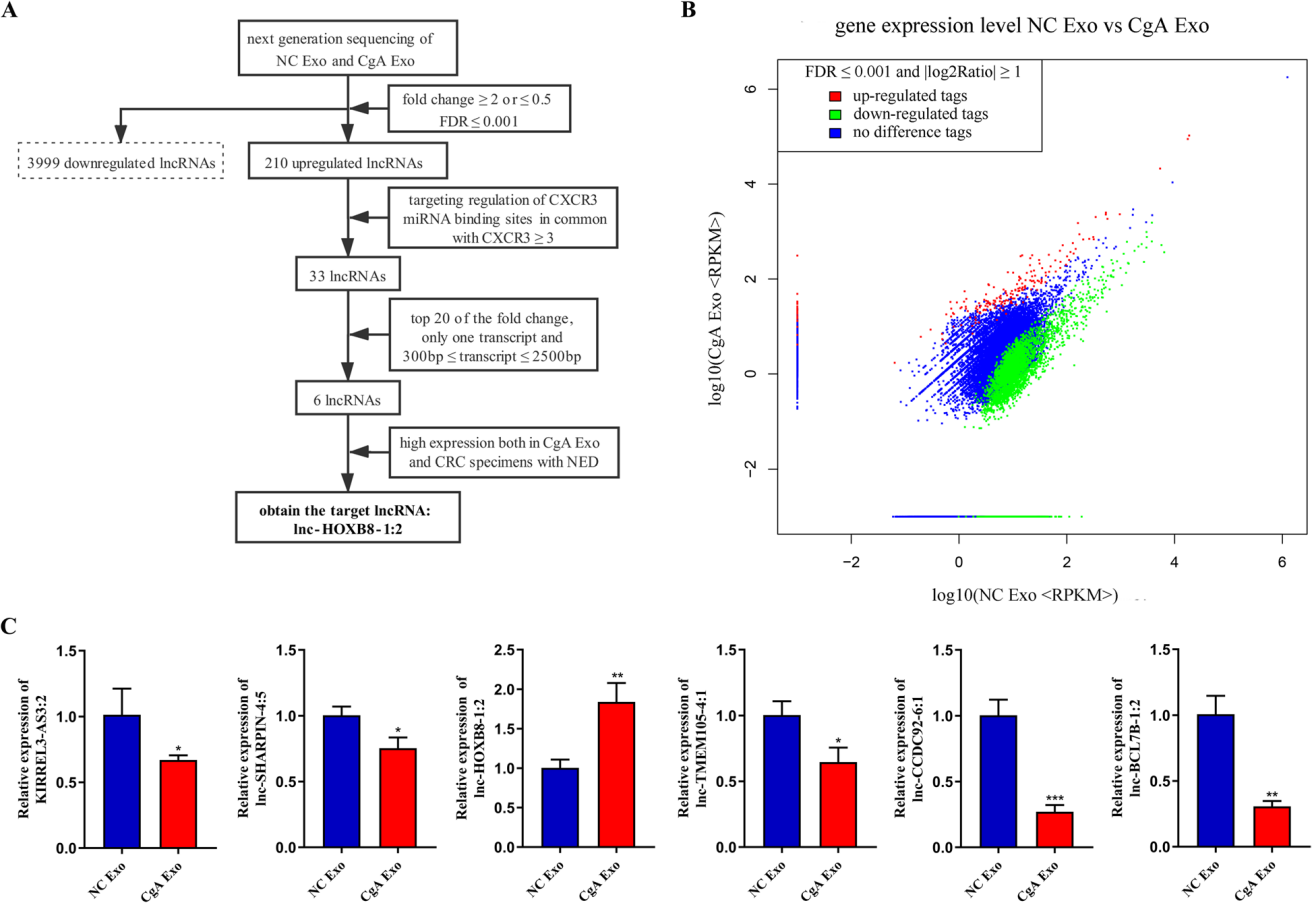
Identification of lnc-HOXB8-1:2 in exosomes. **A**, The diagram of screening target lncRNAs. **B**, Gene distribution map of lncRNAs with a fold change ≥ 2 or ≤ 0.5 and FDR ≤ 0.001 from NC Exo and CgA Exo. log2Ratio, log base 2 of the fold change; |log2Ratio|≥ 1, the fold change ≥ 2 or ≤ 0.5; FDR, false discovery rate; up/downregulated tag, the expression of a gene in CgA Exo was upregulated/downregulated compared to that in NC Exo. **C**, The expression of 6 selected lncRNAs was measured in NC Exo and CgA Exo via qRT–PCR, and lnc-HOXB8-1:2 was highly expressed in CgA Exo. *, *P* < 0.05; **, *P* < 0.01; ***, *P* < 0.001; NC, negative control; CgA, chromogranin A; Exo, exosomes

**Fig. 5:**
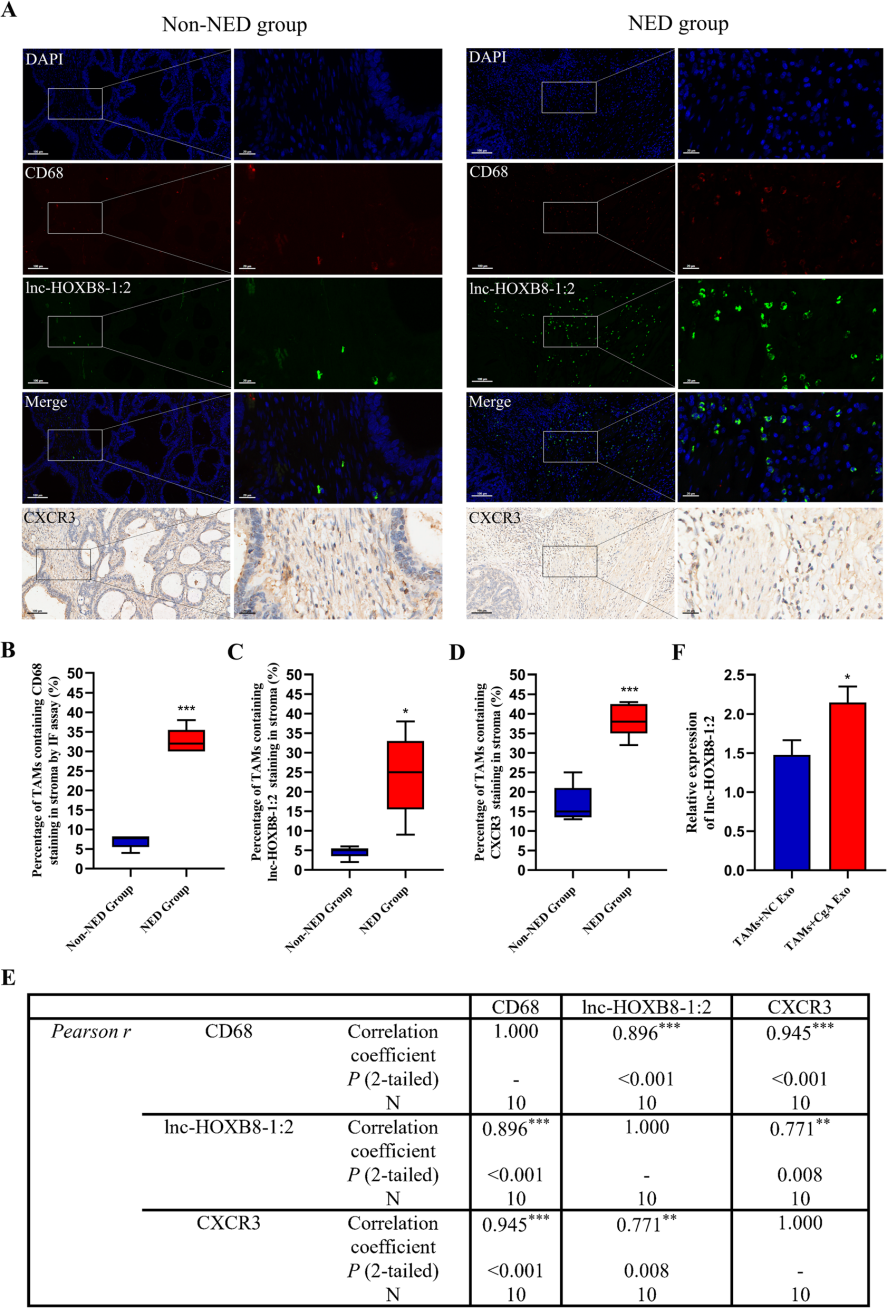
Association between lnc-HOXB8-1:2 and CXCR3 in TAMs. **A**, The rising expression of CXCR3 and lnc-HOXB8-1:2 in TAMs (stained by CD68) that infiltrated neuroendocrine differentiated CRC tissue specimens showed by successive tissue sections. Scale bar =100μm and 20 μm. **B**-**D**, The expression of CD68, lnc-HOXB8-1:2 and CXCR3 in the NED group were significantly upregulated**. E**, Correlation analysis found that a significant positive correlation existed between CD68 and lnc-HOXB8-1:2, CXCR3 and CD68, CXCR3 and lnc-HOXB8-1:2. **F**, qRT–PCR assay revealed an increase of lnc-HOXB8-1:2 in TAMs cocultured with CgA Exo. *, *P* < 0.05; **, *P* < 0.01; ***, *P* < 0.001; TAMs, tumor-associated macrophages; NC, negative control; NED, neuroendocrine differentiation; non-NED, non-neuroendocrine differentiation; CgA, chromogranin A; Exo, exosomes

LncRNAs are believed to serve important roles in tumorigenesis and metastasis, because of alterations in their expression or mutation [18]. We detected lncRNAs in exosomes from LoVo-CgA cells and LoVo-NC cells using next-generation sequencing. Based on the screening criteria mentioned above, 6 lncRNAs (KIRREL3-AS3:2, lnc-SHARPIN-4:5, lnc-HOXB8-1:2, lnc-CCDC92-6:1, lnc-TMEM105-4:1 and lnc-BCL7B-1:2) were selected (Fig. 4A-B). The expression of 6 lncRNAs was detected by qRT–PCR in CgA Exo and NC Exo, showing that lnc-HOXB8-1:2 had remarkably higher expression level in CgA Exo than in NC Exo, while the other selected lncRNAs were expressed at lower levels in CgA Exo (Fig. 4C). To explore the association between lnc-HOXB8-1:2 and CXCR3 in CRC tissue specimens, we evaluated lnc-HOXB8-1:2 and CXCR3 levels in TAMs (stained by CD68) that infiltrated colorectal cancer tissue specimens from the 10 patients mentioned above using FISH assay and IHC and found that the expression of CD68, lnc-HOXB8-1:2 and CXCR3 increased significantly in the same site of the tissue section from the NED group (Fig. 5A-D). The results revealed that CXCR3 expression as well as CD68 expression positively correlated with lnc-HOXB8-1:2 expression in CRC tissue (Fig. 5E). There was also a positive association between CXCR3 expression and CD68 expression (Fig. 5E). Moreover, the lnc-HOXB8-1:2 expression level increased remarkably when TAMs were cocultured with CgA Exo (Fig. 5F).

According to these results, we speculated that exosome-derived lnc-HOXB8-1:2 might induce TAMs to infiltrate neuroendocrine differentiated CRC tissues by modulating CXCR3 expression.

### lnc-HOXB8-1:2 promotes the chemotactic ability of TAMs by regulating the lnc-HOXB8-1:2/hsa-miR-6825-5p/CXCR3 axis

**Fig. 6:**
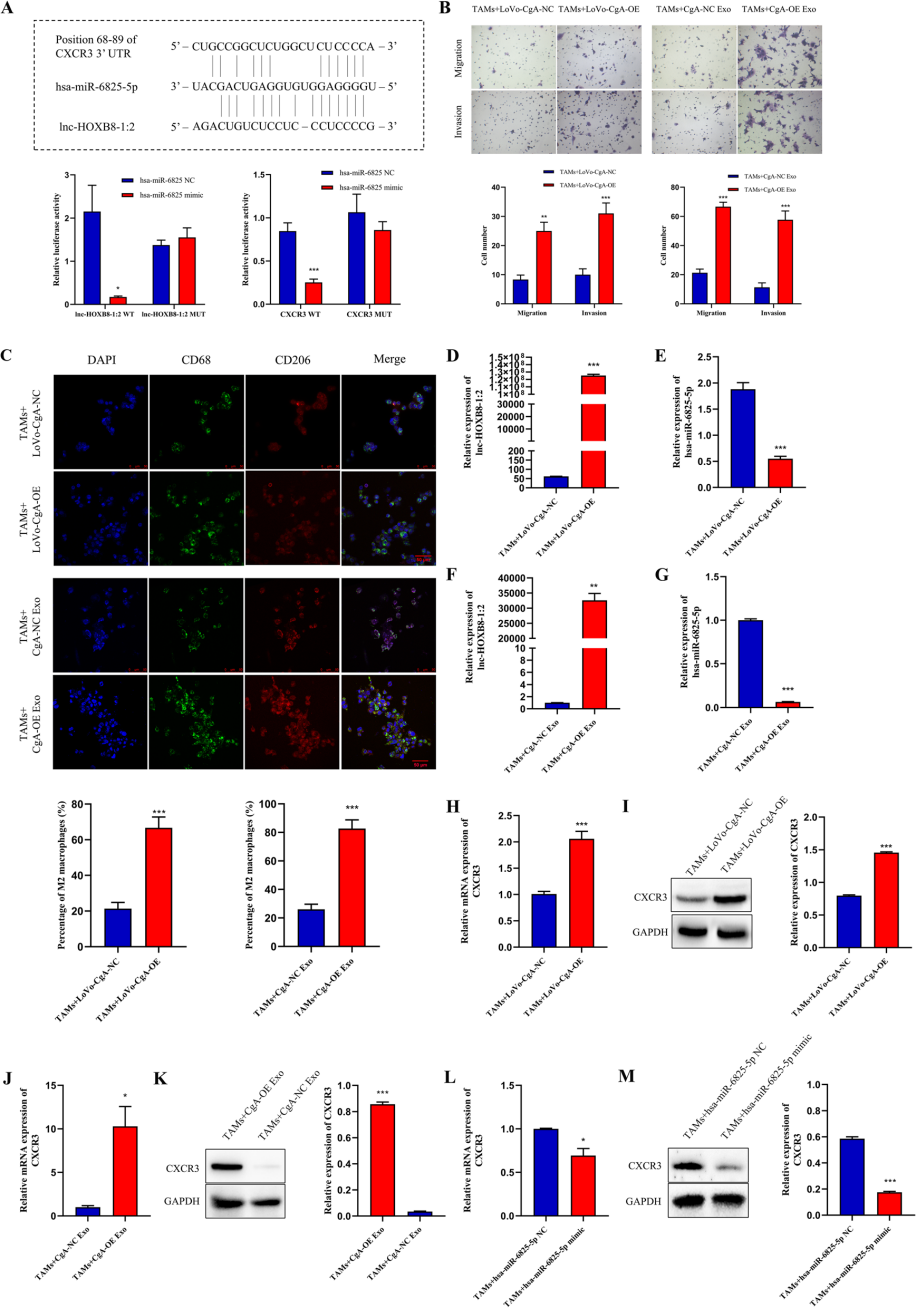
lnc-HOXB8-1:2 induces infiltration and M2 polarization of TAMs by sponging hsa-miR-6825-5p to upregulate CXCR3 expression. **A**, Predicted binding sites among lnc-HOXB8-1:2, hsa-miR-6825-5p and CXCR3 was found using RNA22 database and Target Scan database. And luciferase activity was reduced remarkably in the 293 T cells transfected with the exogenous hsa-miR-6825-5p mimics, and the inhibitory activity of hsa-miR-6825-5p was lost when the binding sites were lost. **B**, TAMs had greater migration and invasion capability after being treated with LoVo-CgA-OE cells or CgA-OE Exo. Scale bar = 200 μm. **C**, The proportion of M2 macrophages (stained by CD68 and CD206) increased when TAMs was cocultured with LoVo-CgA-OE cells or CgA-OE Exo. **D**-**G**, The expression of lnc-HOXB8-1:2 (**D**, **F**) increased while hsa-miR-6825-5p (**E**,**G**) decreased in TAMs cocultured with LoVo-CgA-OE cells or CgA-OE Exo. **H–K**, CXCR3 expression was upregulated in TAMs treated with LoVo-CgA-OE cells or CgA-OE Exo. **L-M**, The CXCR3 expression was reduced in TAMs transfected with the exogenous hsa-miR-6825-5p mimics. **, *P* < 0.01; ***, *P* < 0.001; NC, negative control; OE, overexpress; CgA, chromogranin A; Exo, exosomes; TAMs, tumor-associated macrophages; WT, wild-type; MUT, mutant type

To clarify the functional role of lnc-HOXB8-1:2, the public databases mentioned above were used to predict and validate the target miRNA of lnc-HOXB8-1:2 according to the competitive endogenous RNA (ceRNA) theory [19] (Fig. S1). Data showed that binding sites of hsa-miR-6825-5p were found both on lnc-HOXB8-1:2 and CXCR3 mRNA (Fig. 6A), implying that lnc-HOXB8-1:2 may upregulate CXCR3 expression by sponging hsa-miR-6825-5p. To further confirm this prediction, the dual-luciferase reporter assay was applied to verify the direct interaction among lnc-HOXB8-1:2/hsa-miR-6825-5p/CXCR3 axis. The results showed that overexpression of hsa-miR-6825-5p significantly diminished the luciferase activity of both the lnc-HOXB8-1:2 and CXCR3 vectors including the wild-type binding site but not the mutant binding site (Fig. 6A). To investigated the effect of lnc-HOXB8-1:2 on hsa-miR-6825-5p expression, we constructed a lnc-HOXB8-1:2-overexpressing plasmid and successfully transferred the plasmid into LoVo-CgA cells, and a notable increase in lnc-HOXB8-1:2 was observed both in cells and their secreted exosomes (Fig. S2A-B). The exosomes were also collected from LoVo-CgA-OE cells (CgA-OE Exo) and LoVo-CgA-NC cells (CgA-NC Exo). Then, LoVo-CgA-OE cells, LoVo-CgA-NC cells and corresponding exosomes were cocultured with TAMs separately. According to the transwell assay, the migration and invasion abilities of TAMs were enhanced when they were cocultured with LoVo-CgA-OE cells or CgA-OE Exo (Fig. 6B). Similarly, TAMs tended to differentiate into M2 macrophages after the cocultured with LoVo-CgA-OE cells or CgA-OE Exo (Fig. 6C). Additionally, lnc-HOXB8-1:2 and CXCR3 expression significantly increased in TAMs that were cocultured with LoVo-CgA-OE cells or CgA-OE Exo, while the expression of hsa-miR-6825-5p decreased (Fig. 6D-K). We also transfected has-miR-6825-5p mimics into TAMs (Fig. S3A) and the results displayed that has-miR-6825-5p could significantly inhibit the expression of CXCR3(Fig. 6L-M).

**Fig. 7:**
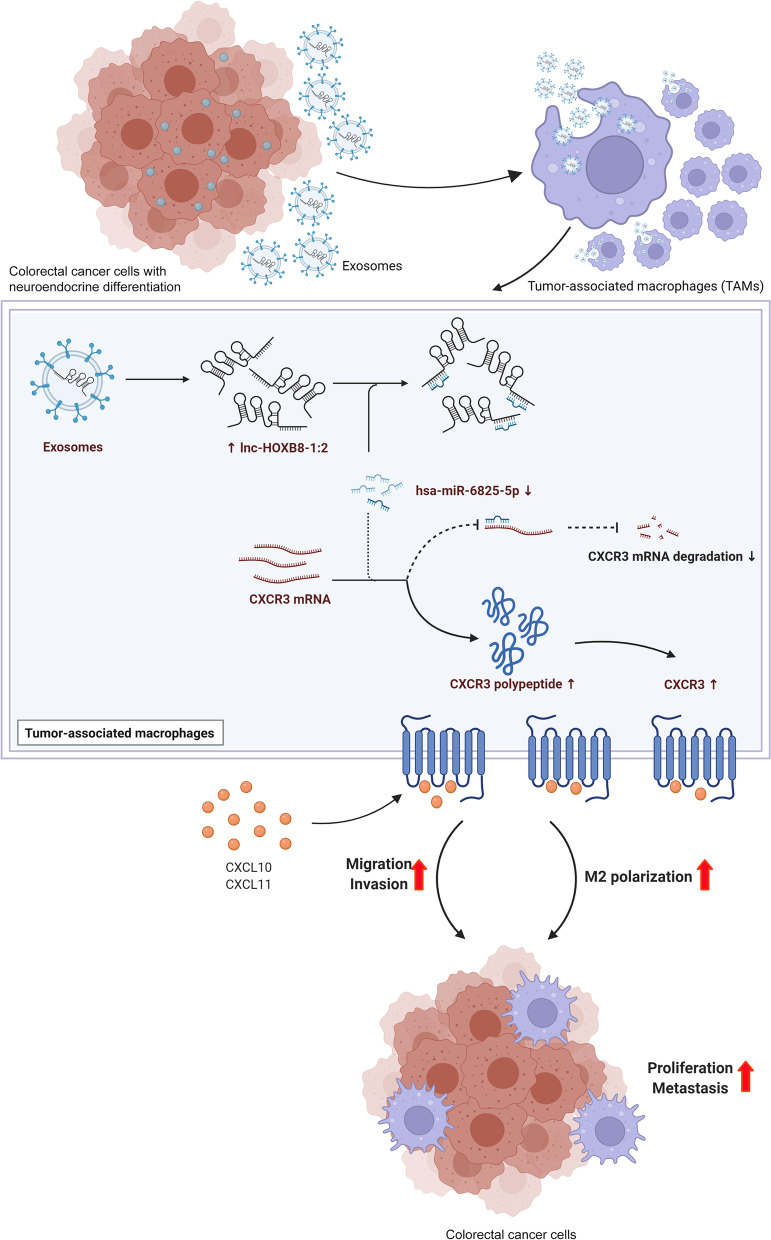
The possible mechanism of lnc-HOXB8-1:2 on promoting CRC progression via lnc-HOXB8-1:2/hsa-miR-6825-5p/CXCR3 axis. (Created with BioRender.com)

These cytological results illustrated that exosome-derived lnc-HOXB8-1:2 induces infiltration and M2 polarization of TAMs by regulating the lnc-HOXB8-1:2/hsa-miR-6825-5p/CXCR3 axis (Fig. 7).

## Discussion

As the crucial mediator of intercellular communication in the tumor microenvironment (TME), exosomes provide cancer cells with the capacity to deliver multiple proteins and nucleic acids to nonneoplastic cells in the TME, and then molecular, transcriptional and translational alterations take place that change the biological behavior of nonneoplastic cells [13, 27]. The reprogrammed nonneoplastic cells start to produce their own exosomes carrying signaling molecules and are delivered back to cancer cells, facilitating their proliferation, as well as immune cells, endothelial cells and fibroblasts in the TME, motivating their tumor-promoting functions [13]. In this study, we confirmed that neuroendocrine differentiated colon cancer cell-derived exosomes with an abundance of lnc-HOXB8-1:2 can be absorbed by tumor-associated macrophages in vitro; thus, the expression of lnc-HOXB8-1:2 increased in TAMs. The function of hsa-miR-6825-5p was suppressed while CXCR3 expression was upregulated so that the invasion and migration of TAMs were enhanced and the M2 polarization of TAMs was stimulated.

Tumor-associated macrophages (TAMs), one of the most abundant immune cells in the TME, acquire different phenotypes in response to diverse growth factors and chemokines released by cancer cells and stromal cells [26]. M1 macrophages play a crucial role in tumoricidal activity, while M2 macrophages are linked to tumor promotion via inhibition of the Th1-like lymphocyte reaction [28, 29]. It was reported that M2 macrophages exert tumor stimulative activities in CRC through regulation of extracellular matrix remodeling, cancer cell metabolism, angiogenesis and the tumor microenvironment [26]. Our previous study also proved that TAMs strengthen the proliferation and invasion of colon cancer cells in vitro [5]. According to the bioinformatics analysis in this study, the infiltration of macrophages, especially M2 macrophages, positively correlates with CgA and CXCR3 gene expression, respectively, in colon adenocarcinoma. However, the results were mixed in rectal adenocarcinoma, probably because of the limited sample size of READ and the different algorithms, both of which contribute to bias. In this study, we also determined that TAMs infiltrating colorectal cancer tissue aggregated around NED cells and that the neuroendocrine differentiated CRC tissue were infiltrated abundant TAMs. Furthermore, lnc-HOXB8-1:2 and CXCR3 levels exhibited positive correlation with the expression of CD68, revealing that both NED and CXCR3 expression were positively associated with the infiltration of TAMs in CRC tissue. Based on these findings, we speculated that CRC cells with NED promote TAM differentiation into M2 macrophages to infiltrate CRC tissue via exosome-derived lnc-HOXB8-1:2, leading to the progression of neuroendocrine differentiated CRC.

The chemokine CXC motif receptor 3 (CXCR3), the CXCL9, CXCL10 and CXCL11 specific receptor, participates in the tumor migration, invasion, angiogenesis and immunity and are mainly expressed on monocytes, effector T cells, NK lymphocytes and cancer cells [24, 30]. In this study, we found CXCR3 expressed in TAMs that infiltrated clinical CRC tissues and were correlated with the presence of neuroendocrine differentiation. Important roles of CXCR3 and its selective ligands have been demonstrated in antitumor functions by mediating immune infiltration [31]. For instance, decreased CXCR3 expression predicted poor prognosis of osteosarcoma and CXCR3 expression was positively associated with immune infiltration of CD8^+^ T cells, M1 macrophages, plasma cells, and activated NK cells [31]. CXCR3^+^ NK-cell and CD8^+^ T-cell tumoral infiltration, directed by CXCL9 and CXCL10, can delay primary tumor growth in MC38 colon carcinoma and TC-1 epithelial carcinoma mouse models when treated with heterodimeric IL-15 [32]. Furthermore, CXCL11 increased CD8^+^ T-cell recruitment to the TME during docetaxel treatment in non-small-cell lung cancer, reducing tumor progression [33]. Nevertheless, this study offered a crucial finding that an increase in CXCR3 expression significantly promotes tumor infiltration of TAMs and M2 polarization in neuroendocrine differentiated CRC in vivo and vitro. A previous paper also showed that CXCL10 and CXCL11 derived from neuroendocrine-like cells induced TAM chemotaxis to CRC cells and enhanced their proliferation and invasion [5]. Collectively, in this study, colon cancer cells with NED released CXCL10 and CXCL11 to recruit CXCR3^+^ TAMs and promote their differentiation into M2 macrophages, favoring tumor progression that demonstrated the tumor-promoting function of CXCR3 and its ligands, CXCL10 and CXCL11, which differed from the findings mentioned above.

However, if CXCR3 plays a vital role in antitumor immune infiltration, why is CXCR3 expression on TAMs increased and correlated with tumor progression in neuroendocrine differentiated colon adenocarcinomas? Competitive endogenous RNA (ceRNA), also-called “miRNA sponge”, is defined as RNA transcripts that compete with endogenous mRNAs for miRNA binding sites (MREs or “miRNA response elements”) via partial sequence complementation to accommodate miRNA expression and functions [19]. The ceRNA theory has been supported in a large proportion of experimental observations. Exosome-derived CircPACRGL manipulated TGF-β expression by sponging miR-142-3p/miR-506-3p to induce N1-N2 neutrophil differentiation, resulting in CRC proliferation and migration [15], whereas lncRNA-PVT1 acted as a competitive inhibitor of miR-455 to downregulate miR-455 and ultimately promote the progression of CRC [34]. Our data showed that lnc-HOXB8-1:2 was abundant in neuroendocrine differentiated colon cancer cell-derived exosomes. By literature review, there were no reports on the functional role and molecular mechanism of lnc-HOXB8-1:2 in colorectal cancer. In our study, lnc-HOXB8-1:2 and CXCR3 mRNA were bioinformatically predicted to have the common binding site of hsa-miR-6825-5p. The dual-luciferase reporter assay provided the evidence that lnc-HOXB8-1:2 directly binding has-miR-6825-5p and CXCR3 was the direct target of has-miR-6825-5p. We also found that increased lnc-HOXB8-1:2 expression was accompanied by decreased hsa-miR-6825-5p expression but upregulation of CXCR3. Overexpression of lnc-HOXB8-1:2 could highlight this phenomenon. Furthermore, has-miR-6825-5p exhibited the carcinogenesis role and partially mediated the oncogenic function of CXCR3 by direct binding. These results suggested that exosome-derived lnc-HOXB8-1:2 acted as a ceRNA of hsa-miR-6825-5p to decrease its level as well as impair its activity so that CXCR3 expression was positively regulated, thus promoting TAM infiltration and M2 polarization and, eventually leading to the advancement of neuroendocrine differentiated CRC.

Another point worth noting was that 6 lncRNAs were selected according to the filtering rules mentioned above, but only lnc-HOXB8-1:2 was highly expressed in exosomes extracted from NED cells, while the others were decreased according to the qRT–PCR assay. RNA sequencing did not provide exact absolute measurements, and due to gene-specific biases, it is acceptable and reasonable that the results of RNA sequencing and qRT–PCR are not identical [35]. More research is needed to confirm whether the other 5 lncRNAs participate in the progression of neuroendocrine differentiated CRC.

To date, the clinical value of neuroendocrine differentiation in CRC remains controversial. Suresh et al. reported that there was no significant correlation between NED and poor prognosis in 53 CRC patients [36], consistent with the study by Cho et al. [37]. In contrast, the research of Chen et al. found that neuroendocrine differentiated CRC was prone to lymph node metastasis [38]. Guo et al. suggested that the overall survival of patients with neuroendocrine differentiated CRC was less favorable and associated with positive lymph nodes [39]. Similarly, our study indicated that the presence of NED predicted unfavorable prognosis of CRC patients, such as shorter overall survival and higher risk of progression. In this study, we further illustrated that exosome-derived lnc-HOXB8-1:2 induced the migration and invasion of TAMs to promote the development of colon adenocarcinoma with neuroendocrine differentiation via sponging hsa-miR-6825-5p to upregulate CXCR3 expression. These results add to the evidence that neuroendocrine differentiation is a risk factor for poor prognosis in CRC patients with NED and provide a promising target in CRC therapy.

## Conclusion

In summary, we first reported the existence of lnc-HOXB8-1:2 in neuroendocrine differentiated CRC-derived exosomes, and lnc-HOXB8-1:2 acted as a ceRNA competitively binding hsa-miR-6825-5p to regulate the lnc-HOXB8-1:2/hsa-miR-6825-5p/CXCR3 axis, leading to the infiltration and M2 polarization of TAMs, which serves an oncogenic role in CRC progression. This study helps us to understand on the mechanism of exosomes and lncRNAs in CRC development, offering a potential targeted therapy against CRC.

## Supplementary Information

**Additional file 1: Supplementary Tables.**

**Additional file 2: Figure S1.**

**Additional file 3: Figure S2.**

**Additional file 4:Figure S3.**

**Additional file 5: **Full-length gels and blots.

## Data Availability

lncRNA sequences and miRNA sequence used during the current study are available in the LNCipedia database (https://lncipedia.org/) and miRbase database (https://mirbase.org/). The data of the gene expression level of CgA and CXCR3 and the infiltration of macrophages are available in the Timer 2.0 database (http://timer.cistrome.org/). The other data used and/or analyzed during the current study are available from the corresponding author on reasonable request.
