# Supplementary material for: Exosome-derived lnc-HOXB8-1:2 induces tumor-associated macrophage infiltration to promote neuroendocrine differentiated colorectal cancer progression by sponging hsa-miR-6825-5p
Source: BMC Cancer. 2022 Aug 27;22:928. doi: 10.1186/s12885-022-09926-1 (PMC9419355; doi:10.1186/s12885-022-09926-1)
Supplement: Supplementary file 1 — Additional file 1: Supplementary Tables. [file 12885_2022_9926_MOESM1_ESM.docx]

**Supplementary tables**

**Table S1** The catalog number of main reagents.

| Reagent | Company | Product code | Production place |
| --- | --- | --- | --- |
| DMEM-12 | Gibco | 11330032 | USA |
| 0.25% Trypsin-EDTA | Gibco | 25200072 | USA |
| RPMI-1640 | Gibco | 11875119 | USA |
| FBS | Gibco | 16000044 | USA |
| 1×PBS (pH 7.4) | Gibco | C10010500BT | USA |
| G418 | MP Biomedicals | 0215878291 | Germany |
| 4% Paraformaldehyde | Beyotime | P0099 | China |
| PMA | Sigma | P8139 | USA |
| Lipofectamine 2000 | Invitrogen | 11668027 | USA |
| Lipofectamine RNAimax | Invitrogen | 13778030 | USA |
| IL-4 | Sigma | SRP3093 | USA |
| RIPA | Thermo Scientific | 89900 | USA |
| Matrigel | BD | 354234 | USA |
| Crystal Violet Staining Solution | Beyotime | C0121 | China |
| SDS | Thermo Scientific | 28365 | USA |
| Tris | Servicebio | GC208001 | China |
| PVDF membrane | BIO-RAD | 1620177 | China |
| 20×SSC (pH 7.0) | Servicebio | G3015 | China |
| Non-fat milk | Nestle | 1010708 | China |
| TRIzol | MRC | TR118-500 | USA |
| M-MLV Reverse Transcriptase | Promega | M1705 | USA |
| DAPI | Abcam | ab228549 | USA |
| Total Exosome Isolation Reagent (from cell culture media) | Thermo Scientific | 4478359 | USA |
| Total Exosome RNA and Protein Isolation Kit | Life Technologies | 4478545 | USA |
| Qubit® RNA HS Assay Kit | Life Technology | Q32852 | USA |
| Bicinchoninic Acid (BCA) Protein Quantification Kit | Sigma | BCA1-1KT | USA |
| GoTaq® RT-qPCR Master Mix | Promega | A6020 | USA |
| Enhanced Chemiluminescence (ECL) Kit | Forevergen | YNSJ24 | China |
| PKH67 Green Fluorescent Cell Linker Mini Kit | Sigma | MINI67 | USA |
| Dual-Luciferase™ Reporter (DLR™) Assay Systems | Promega | E1910 | USA |

**Table S2** The catalog number of main antibodies.

| Antibody | Company | Product code | Production place |
| --- | --- | --- | --- |
| Anti-Chromogranin A Antibody | Abcam | ab283265 | USA |
| Anti-Synaptophysin Antibody | Abcam | ab32127 | USA |
| Anti-CD68 Antibody (KP1) | Santa Cruz | sc-20060 | USA |
| CXCR3B-Specific Monoclonal Antibody | Proteintech | 60065-1 | USA |
| Anti-CD63 Antibody | Abcam | ab134045 | USA |
| HSPA8 (D12F2) Rabbit mAb | CST | 8444 | USA |
| Anti-TSG101 Antibody | Abcam | ab125011 | USA |
| Anti-ALIX Antibody | Abcam | ab275377 | USA |
| Anti-GAPDH Antibody-Loading Control | Abcam | ab9485 | USA |
| HRP-linked polyclonal rabbit anti-mouse IgG | Boster | SV0004 | USA |
| CD68 (D4B9C) XP® Rabbit mAb | CST | 76437 | USA |
| CD206/Mannose Receptor/MMR Ab | Santa Cruz | sc-58986 | USA |
| Alexa Fluor 488-Donkey anti-Rabbit IgG (H+L) Cross-Adsorbed Secondary Antibody | Invitrogen | A21206 | USA |
| Alexa Fluor 546-Goat anti-Mouse IgG (H+L) Highly Cross-Adsorbed Secondary Antibody | Invitrogen | A11040 | USA |
| Alexa Fluor 594-Goat anti-Rabbit IgG (H+L) Cross-Adsorbed Secondary Antibody | Invitrogen | A11012 | USA |

**Table S3** Sequences of qRT-PCR primers

| Gene | Forward primer (5’ to 3’) | Reverse primer (5’ to 3’) |
| --- | --- | --- |
| CgA | CCACCGCTCCTGCCAC | CAGCTGGCGGTGTGGG |
| CXCR3 | GCTCTGAGGACTGCACCATTG | TGAAGTTTTAGTTTCCAAATGAGAAGGG |
| KIRREL3-AS3:2 | TTCTGGGTTTCTGCACATCC | TCACAGGACCCAGCACTTG |
| lnc-SHARPIN-4:5 | AACTTGACGGAGAGAGAAGAGC | GCAGCGTCTTCAAGTGTGAC |
| lnc-HOXB8-1:2 | TTCTGGCACCTCTGAATTCTCC | ACACAGACGCAGAACACAAG |
| lnc-CCDC92-6:1 | ACCCCTCAGCCTTCTCCTAC | ACCGACATTCCTTGGGAGATG |
| lnc-TMEM105-4:1 | TGAGGCGGCCAGGAAGT | CTTGTAGAAGCTCCAGATCCTGGA |
| lnc-BCL7B-1:2 | TCAGCTTAAGGTGGACAGCAG | AGGTTCATCAGCAACTTCCG |
| hsa-miR-6825-5p | TGGGGAGGTGTGGAGTCA | GTCGTATCCAGTGCAGGGTCCGAGGTATTCGCACTGGATACGACATGCTG |
| GAPDH | AGCCACATCGCTCAGACAC | GAATTTGCCATGGGTGGA |

**Table S4** FISH sequences

| Name | Sequence |
| --- | --- |
| lnc-HOXB8-1:2 (FISH) | TCTGTATGGTGCCAAAGGACACCCCAAAGCCATTCCCATAGATACCAGCTGTGTCTTTCCGAGGGCTCCCCAAAGGATAAGTCAACTTTCAGAGGCTCAGCACAGACACCCATGGCCGGGGTGCTGGTGCCGCTCATACACTAACTAGCTGAGATGATGGTCACTCCGTGAGTAAAGGCCAGTTAGGCAAC |

**Table S5** Sequence containing the wild-type or mutant binding sites with hsa-miR-6825-5p

| Name | Sequence |
| --- | --- |
| lnc-HOXB8-1:2 WT | CTCGAGGAATCTTGTTCAGCGCGGACTCAACGCCAGGGCGCCGCCTAGAGGTTGGTCTCTGTCTCGGCCTCACCCGCCGGGAGACCACAGAGCTGCTTCCCCAGCCGCCCGCCGCCAGAAATTGGAAAAAAAAAAAAAAAAAAAAAAAAAATCCAGCTGGGGTCTAGGAACTCGGCTTCTGGCACCTCTGAATTCTCCGAGACTGTCTCCTCCCTCCCCGCCTGTAATGAACCCTGTGAAGGGAGACAGGCCAGGAAGTCCCAGAAATATTTATTCTTGTGACTCTCACAAAATGGAAAAGGGTCTCAATTTTTGTTTCTTTAAGGAACTTGTGTTCTGCGTCTGTGTCTACACTGCCTCCTCTCACCAACCAAATTGTCTAGCCCCCCTCCAGTTACGCTAGAACTCTGCTTTAGCGGCCGC |
| lnc-HOXB8-1:2 MUT | CTCGAGGAATCTTGTTCAGCGCGGACTCAACGCCAGGGCGCCGCCTAGAGGTTGGTCTCTGTCTCGGCCTCACCCGCCGGGAGACCACAGAGCTGCTTCCCCAGCCGCCCGCCGCCAGAAATTGGAAAAAAAAAAAAAAAAAAAAAAAAAATCCAGCTGGGGTCTAGGAACTCGGCTTCTGGCACCTCTGAATTCTCCGAGACTGTGAGGAGGGACCCCGCCTGTAATGAACCCTGTGAAGGGAGACAGGCCAGGAAGTCCCAGAAATATTTATTCTTGTGACTCTCACAAAATGGAAAAGGGTCTCAATTTTTGTTTCTTTAAGGAACTTGTGTTCTGCGTCTGTGTCTACACTGCCTCCTCTCACCAACCAAATTGTCTAGCCCCCCTCCAGTTACGCTAGAACTCTGCTTTAGCGGCCGC |
| CXCR3 3'UTR WT | CTCGAGGGCCGGAATCCGGGCTCCCCTTTCGCCCACAGTCTGACTTCCCCGCATTCCAGGCTCCTCCCTCCCTCTGCCGGCTCTGGCTCTCCCCAATATCCTCGCTCCCGGGACTCACTGGCAGCCCCAGCACCACCAGGTCTCCCGGGAAGCCACCCTCCCAGCTCTGAGGACTGCACCATTGCTGCTCCTTAGCTGCCAAGCCCCATCCTGCCGCCCGAGGTGGCTGCCTGGAGCCCCACTGCCCTTCTCATTTGGAAACTAAAACTTCATCTTCCCCAAGTGCGGGGAGTACAAGGCATGGCGTAGAGGGTGCTGCCCCATGAAGCCACAGCCCAGGCCTCCAGCTCAGCAGTGACTGTGGCCATGGTCCCCAAGACCTCTATATTTGCTCTTTTATTTTTATGTCTAAAATCCTGCTTAAAACTTTTCAATAAACAAGATCGTCAGGAGCGGCCGC |
| CXCR3 3'UTR MUT | CTCGAGGGCCGGAATCCGGGCTCCCCTTTCGCCCACAGTCTGACTTCCCCGCATTCCAGGCTCCTCCCTCCCTCTGCCGGCTCTGGCTGAGGGGTATATCCTCGCTCCCGGGACTCACTGGCAGCCCCAGCACCACCAGGTCTCCCGGGAAGCCACCCTCCCAGCTCTGAGGACTGCACCATTGCTGCTCCTTAGCTGCCAAGCCCCATCCTGCCGCCCGAGGTGGCTGCCTGGAGCCCCACTGCCCTTCTCATTTGGAAACTAAAACTTCATCTTCCCCAAGTGCGGGGAGTACAAGGCATGGCGTAGAGGGTGCTGCCCCATGAAGCCACAGCCCAGGCCTCCAGCTCAGCAGTGACTGTGGCCATGGTCCCCAAGACCTCTATATTTGCTCTTTTATTTTTATGTCTAAAATCCTGCTTAAAACTTTTCAATAAACAAGATCGTCAGGAGCGGCCGC |
