# Supplementary figures and images for: Exosome-derived lnc-HOXB8-1:2 induces tumor-associated macrophage infiltration to promote neuroendocrine differentiated colorectal cancer progression by sponging hsa-miR-6825-5p
Source: BMC Cancer. 2022 Aug 27;22:928. doi: 10.1186/s12885-022-09926-1 (PMC9419355; doi:10.1186/s12885-022-09926-1)

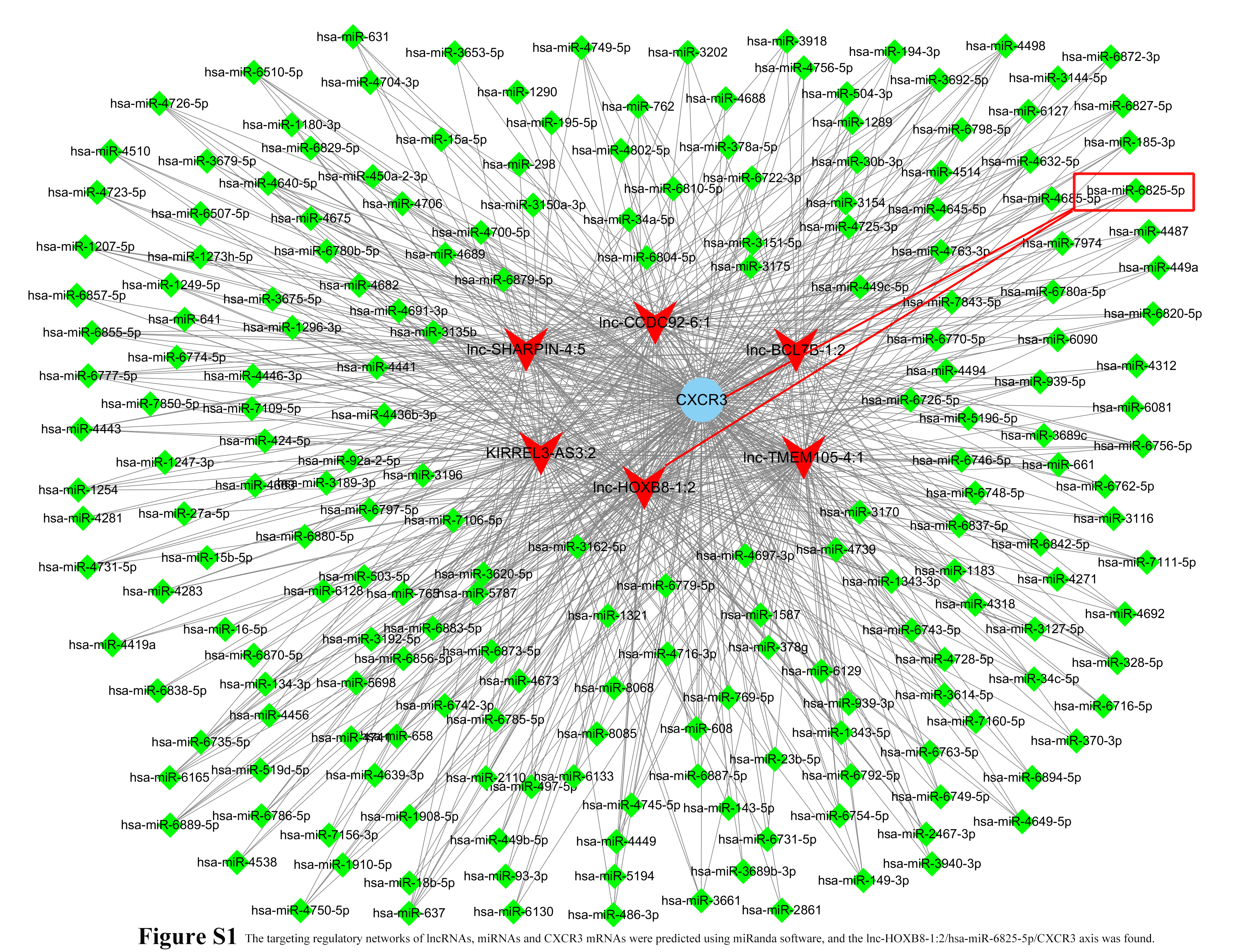

Supplement: Supplementary file 2 — Additional file 2: Figure S1. [file 12885_2022_9926_MOESM2_ESM.tif]

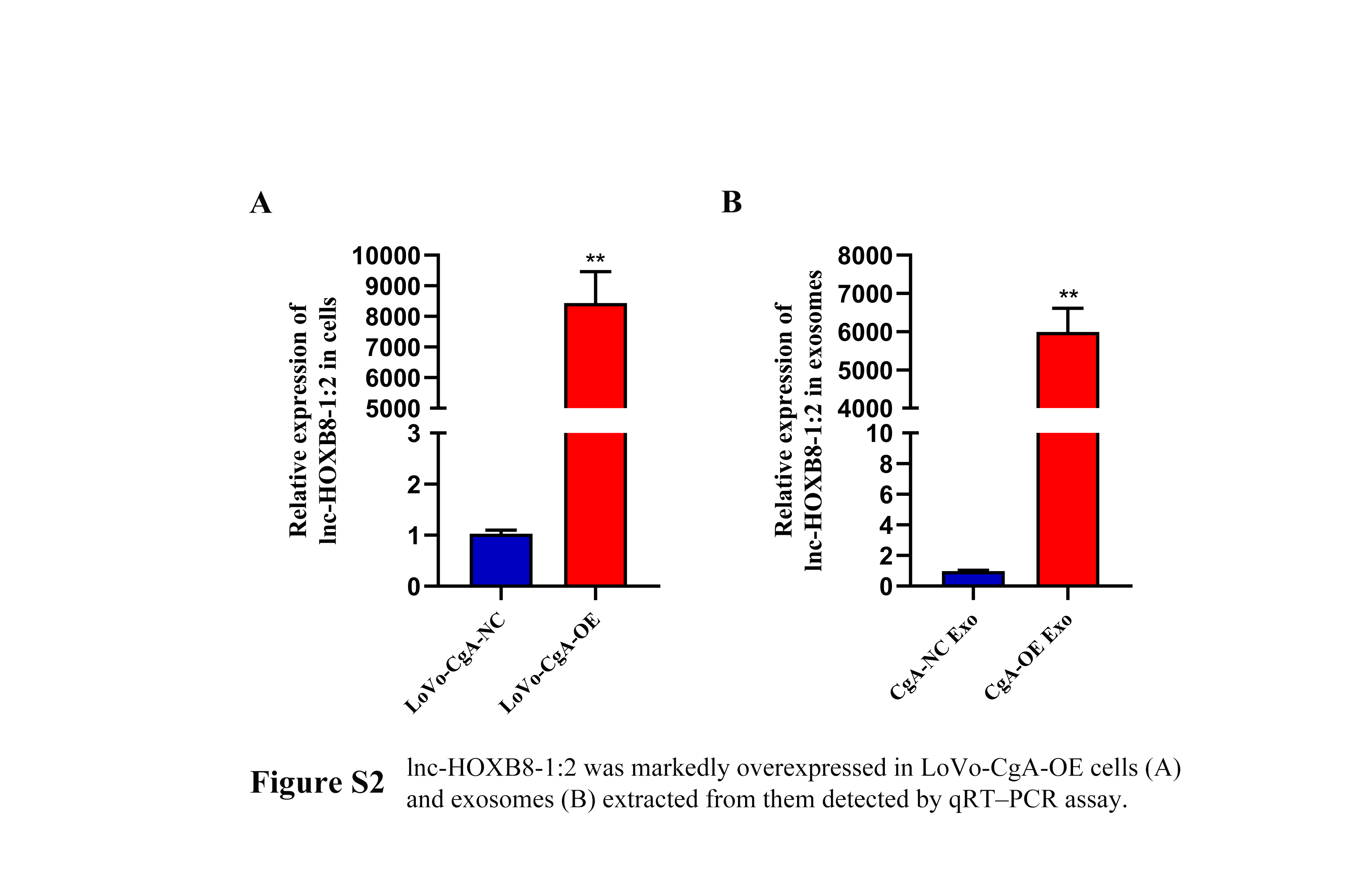

Supplement: Supplementary file 3 — Additional file 3: Figure S2. [file 12885_2022_9926_MOESM3_ESM.tif]

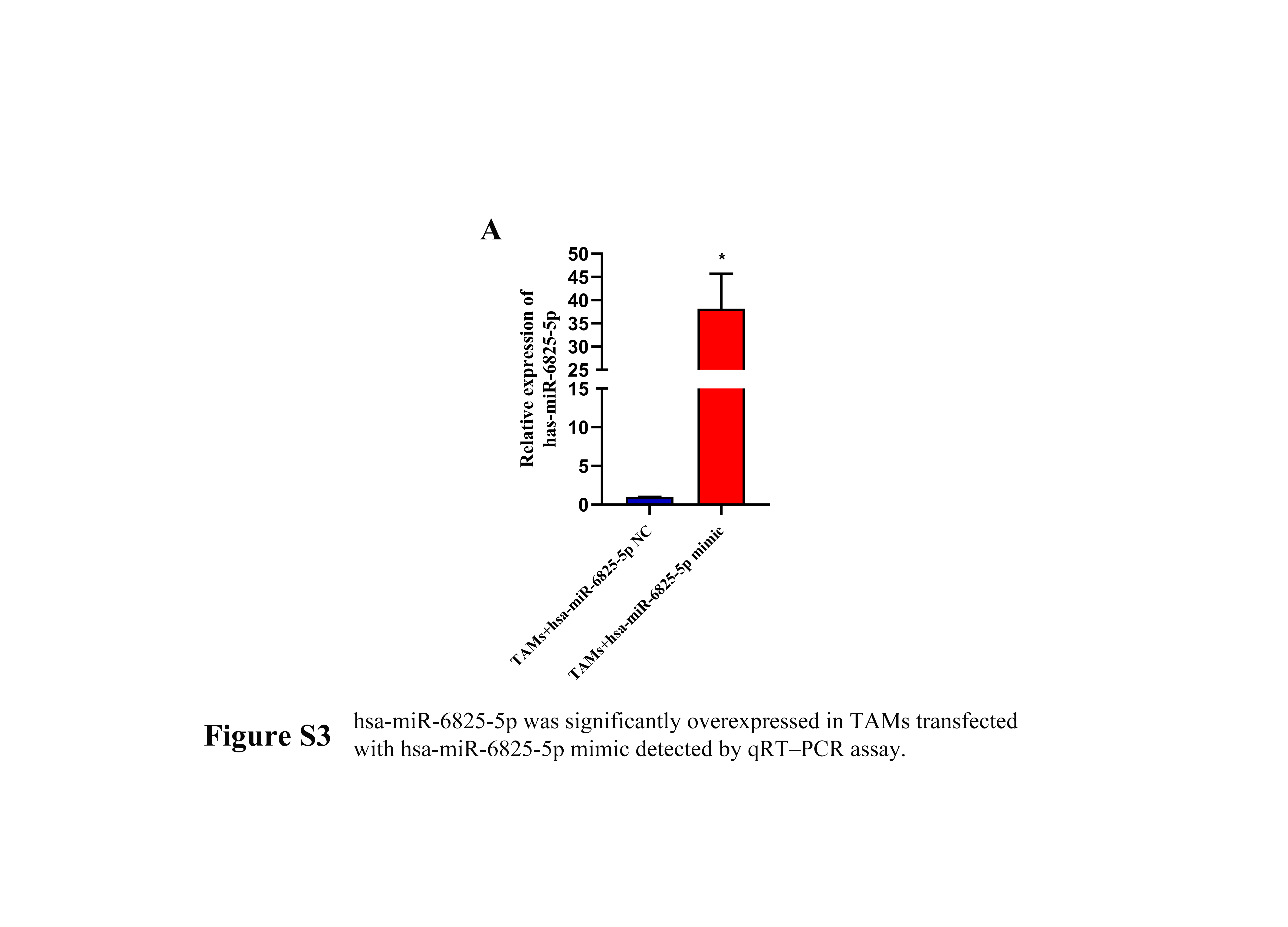

Supplement: Supplementary file 4 — Additional file 4:Figure S3. [file 12885_2022_9926_MOESM4_ESM.tif]

Full-length gels and blots

Figure 2C

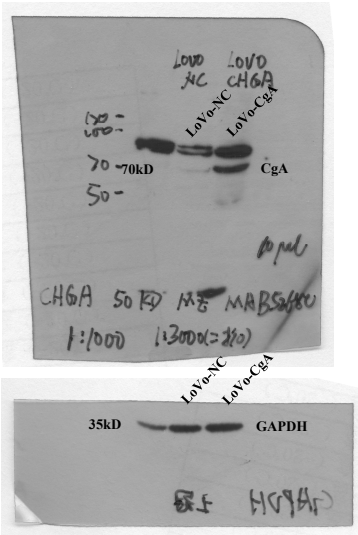

Figure 2F

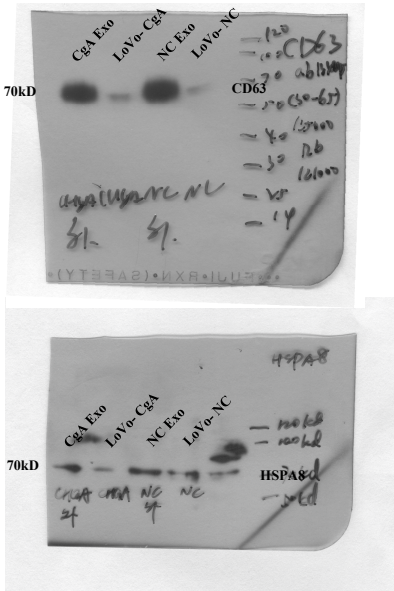

Figure 3E

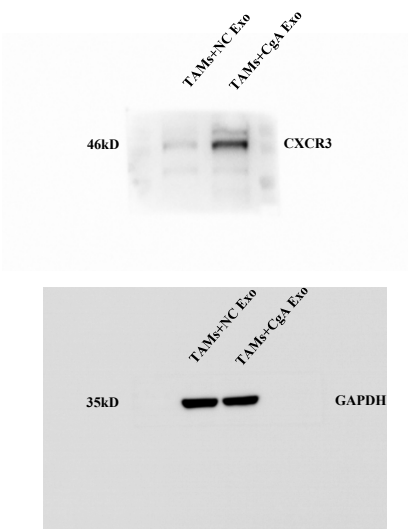

Figure 6I

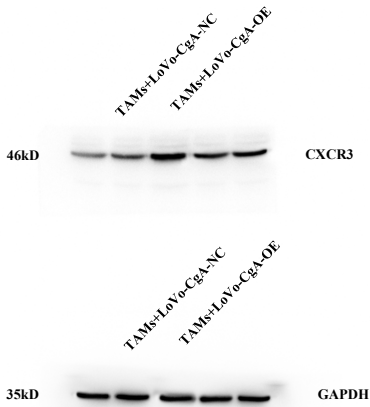

Figure 6K

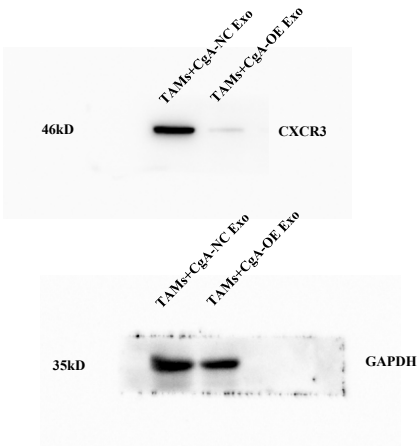

Figure 6M

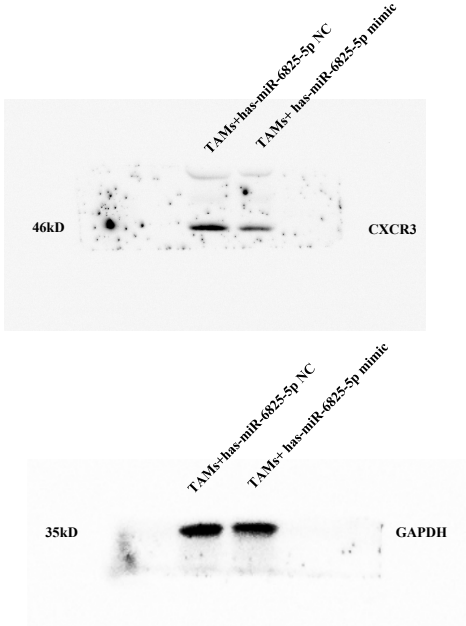

Supplement: Supplementary file 5 — Additional file 5: Full-length gels and blots. [file 12885_2022_9926_MOESM5_ESM.pdf]
